# Extraction and Reimplantation of a Subcutaneous Implantable Cardioverter Defibrillator: Two Cases and a Review of the Literature

**DOI:** 10.7759/cureus.67737

**Published:** 2024-08-25

**Authors:** Cyrus Moini, Jaouad Nguadi, Djamila Rahim, Audrey Lefoulon, Damien Poindron, Antonio Fiore, Thierry Folliguet, Nicolas Lellouche

**Affiliations:** 1 Cardiology, Groupe Hospitalier Sud Ile de France, Melun, FRA; 2 Cardiology, Antony Private Hospital, Antony, FRA; 3 Cardiology, Les Fontaines Clinic, Melun, FRA; 4 Cardiac Surgery, Henri Mondor University Hospital, Creteil, FRA; 5 Cardiology, Henri Mondor University Hospital, Creteil, FRA

**Keywords:** traction, lead extraction, infection, lead fracture, subcutaneous implantable cardioverter defibrillator

## Abstract

For several years, implantable cardioverter defibrillators (ICDs) have been the cornerstone for the prevention of sudden cardiac death. However, the weakness of traditional transvenous ICD systems lies in the intravascular lead, which is prone to issues such as conductor fracture, insulation abrasion, risk of dislodgement, and infection. With the new generation of subcutaneous defibrillators, these risks are far less common. To date, the frequency of lead fracture is very low, and infection is much rarer. The management of these complications requires complete lead extraction. Traction is the reference procedure, sometimes necessitating the use of a dilating sheath. These techniques remain straightforward to perform without significant risk of procedural complications. Nevertheless, they must be carried out by an expert in cardiac pacing. We report here two cases with indications for lead extraction: one for lead dysfunction and the other for an infection related to a replacement procedure. The management approaches will be described, followed by a review of the literature.

## Introduction

The implantable cardioverter defibrillator (ICD) is effective in preventing sudden cardiac death in patients with life-threatening ventricular arrhythmia. The weak point of traditional ICD systems is the transvenous lead (TVL), which is prone to issues such as conductor fracture, insulation abrasion, extraction risks, and infection. Complications related to TVL can reach 20% in leads implanted over a decade, especially in young and active patients [1]. To address these concerns, the subcutaneous ICD (S-ICD) was developed.

However, in December 2020, Boston Scientific issued a safety notice regarding the risk of body lead fracture in the subcutaneous electrode, leading to an FDA class I recall [2]. To date, 68 cases of lead fracture have been reported globally, with an estimated occurrence of approximately 0.31% at 75 months, likely underestimated [3]. Fractures usually occur immediately after the distal part of the proximal electrode, where adhesive is used during assembly to connect the conductor to the proximal sensing electrode [4,5]. These fractures show similar primary and secondary detection vectors, a flat alternate vector, and system impedance exceeding 400 ohms, resulting in beeping tones from the ICD. If the vector is programmed for secondary or alternate sensing, warning or precursor signals of the fracture may be stored, indicating a non-physiological mechanical artifact.

Rare cases of infection during new implantation or S-ICD replacement have also been reported. Lead issue management is similar regardless of the extraction indication.

We report two cases with lead extraction indications: one for lead malfunction and the other for infection related to a replacement procedure. We describe the associated clinical management and review the literature for global indications of S-ICD extraction.

## Case presentation

Case 1

**Figure 1:**
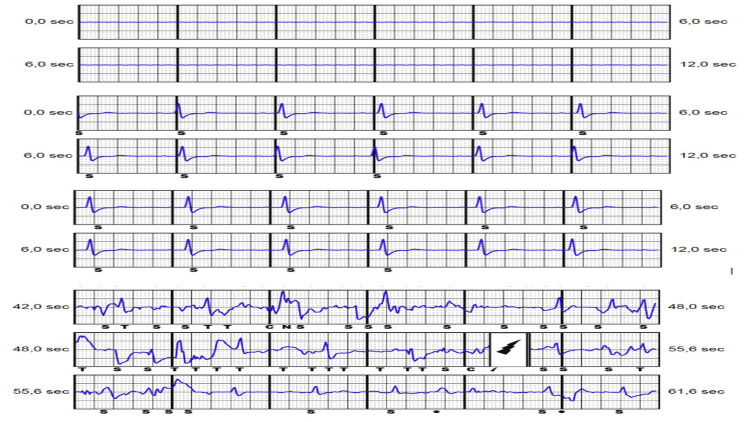
(A) Flat alternate vector, (B) primary vector, and (C) secondary vector, similar to the primary vector. (D) Event classified as VT with delivered therapy showing mechanical artifacts.

**Figure 2:**
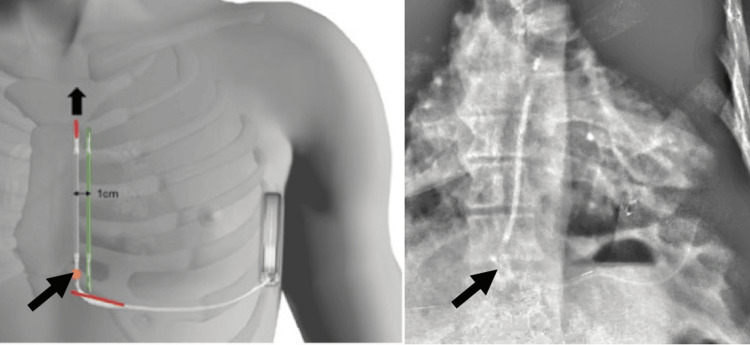
Fracture point of the lead, distal to the proximal electrode. In red: The two incisions made. Arrow: Direction of extraction through the supplementary distal incision. In green: New tunneling 1 cm away from the initial lead.

**Figure 3:**
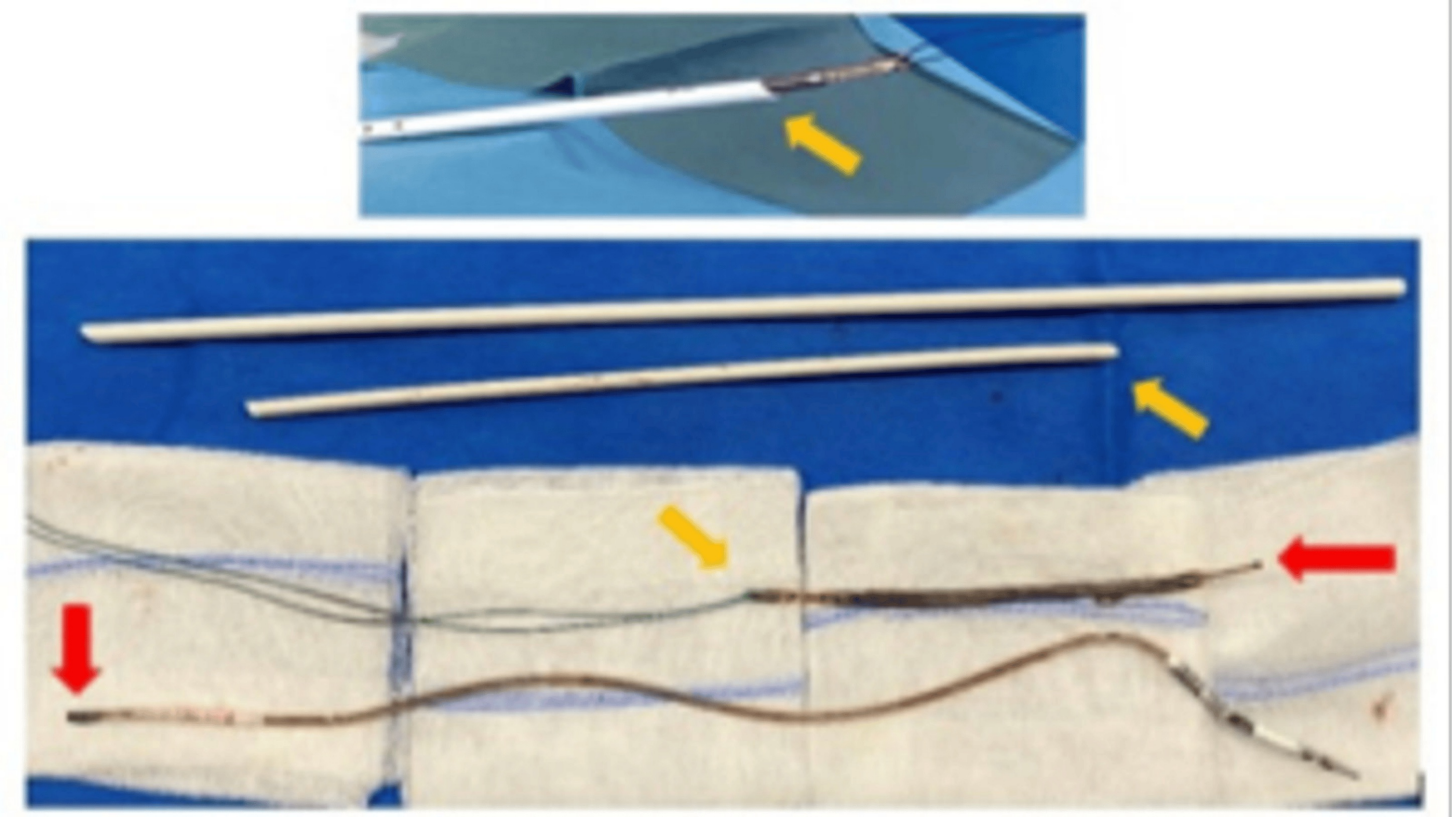
Top: Extracted lead with the dilator sheath through the distal incision. Bottom: Lead extracted in two pieces with the dilator sheath. Red arrows indicate the fracture point. Orange arrows indicate the direction of extraction.

In July 2020, a 58-year-old man with ischemic cardiomyopathy underwent secondary prevention implantation of an S-ICD. Twenty-three months later, the S-ICD delivered a shock at night, prompting a follow-up call. Examination revealed elevated electrode impedance (>400 ohms) and three episodes classified as “ventricular tachycardia,” with one treated. All three events exhibited mechanical artifacts. Subcutaneous electrocardiogram (S-ECG) analysis revealed identical primary and secondary vectors and a flat alternate vector (Figure 1). Chest X-rays confirmed lead rupture at the left xypho-sternal angle. The patient, although asymptomatic, denied any chest trauma. Through a xyphoid incision, a clear fracture was observed immediately after the distal part of the proximal electrode. The proximal lead section was successfully removed through simple traction without complications. To prevent partial extraction risks, an additional incision distal to the lead was made [5]. A Cook dilator sheath was used and adjusted to the remaining lead length. A sturdy thread was inserted into the lead’s end hole and secured in the extraction sheath. Adhesion separation involved a push-pull motion between the lead and the sheath, complemented by clockwise and counterclockwise rotations of the sheath. The new device was implanted on the left parasternal side (Figures 2, 3). The system underwent successful testing, and X-ray follow-up confirmed proper device positioning. Front and left profile chest X-rays confirmed lead rupture at the left xypho-sternal angle, consistent with descriptions in the literature. The patient, although asymptomatic, denied any chest trauma.

The system underwent successful testing (65J, 74 ohms, time to treatment: 17 s). The X-ray follow-up confirmed proper device positioning.

Case 2

**Figure 4:**
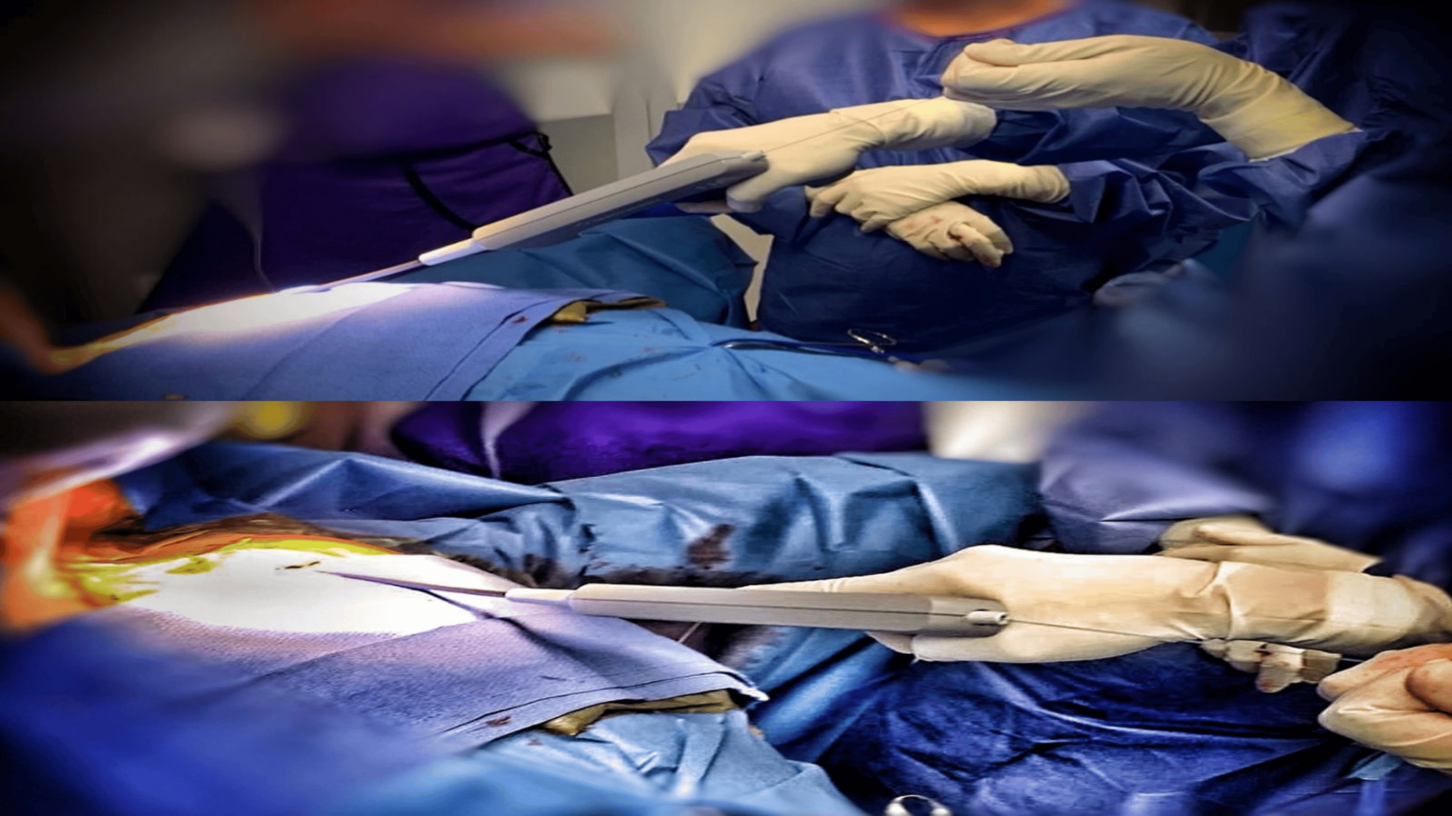
Images of the Spectranetics TightRail progression with continuous tension on the wire.

In September 2023, a 75-year-old man with ischemic cardiomyopathy underwent an S-ICD replacement due to normal battery depletion. The situation was complicated by poorly controlled diabetes and significant social difficulties, leading to neglect of post-operative care. This patient required a complete S-ICD system extraction due to a pocket infection following the generator change. The device extraction, pocket management, and lead access between the xiphoid incision and its proximal part were straightforward. However, parasternal access to the lead was challenging due to fibrosis six years after the initial implantation. To prevent any lead fracture with a single traction method, specific equipment was used: a sheath and then Spectranetics (Figure 4), and the first attempt was successful, with a total hospitalization duration of five days. The patient was discharged with antibiotic treatment and a Zoll LIFE vest for six weeks, awaiting a new intervention with a transvenous ICD system.

## Discussion

In our clinical experience, the first instance of an incomplete fracture in an S-ICD lead occurred in 2020 due to mechanical trauma, unrelated to any recall issues [6]. The literature also describes cases of complete fractures without evident mechanical impact, similar to our findings.

It is crucial to scrutinize lead failure rates in publications, especially since the Model 3501 S-ICD electrode has been in distribution since June 2017, with some studies involving the previous model (ref. 3401), which is not subject to recall.

For example, the Praetorian randomized study [7], conducted from March 2011 to January 2017 (using Model 3401), demonstrated the S-ICD system’s non-inferiority compared to transvenous ICDs. A component of the composite primary endpoint included lead replacements. Given a stable failure rate around 0.3% in recalled populations, the benefit-risk ratio remains favorable for the fully subcutaneous approach.

Regarding transvenous lead data, individuals at risk of S-ICD lead fractures often include young or athletic patients, especially those participating in high-impact sports. Manufacturers recommend weekly remote monitoring and quarterly face-to-face check-ups. Additionally, chest X-rays remain pivotal for confirming suspicions (both lateral and postero-anterior views).

Managing these failures typically involves safe and straightforward extraction procedures. A recent Italian review validates these findings from a pooled analysis of 30 studies encompassing 207 extractions [8].

Our own procedures have shown that using a sheath for extraction has been uncomplicated. The decision to re-implant slightly leftward by 1 cm has proven successful and provided a viable alternative. Since July 2021, an improved subcutaneous lead version has been available, with no reported fractures post-implementation. Literature indicates few instances where rotating mechanical dilator sheaths were employed. Patel et al. first reported success using a Spectranetics TightRail for subcutaneous S-ICD lead extraction, followed by Allison et al., who utilized both TightRail and the Cook Medical Bulldog lead extender [9,10].

However, extended follow-up is necessary to validate these findings.

**Figure 5:**
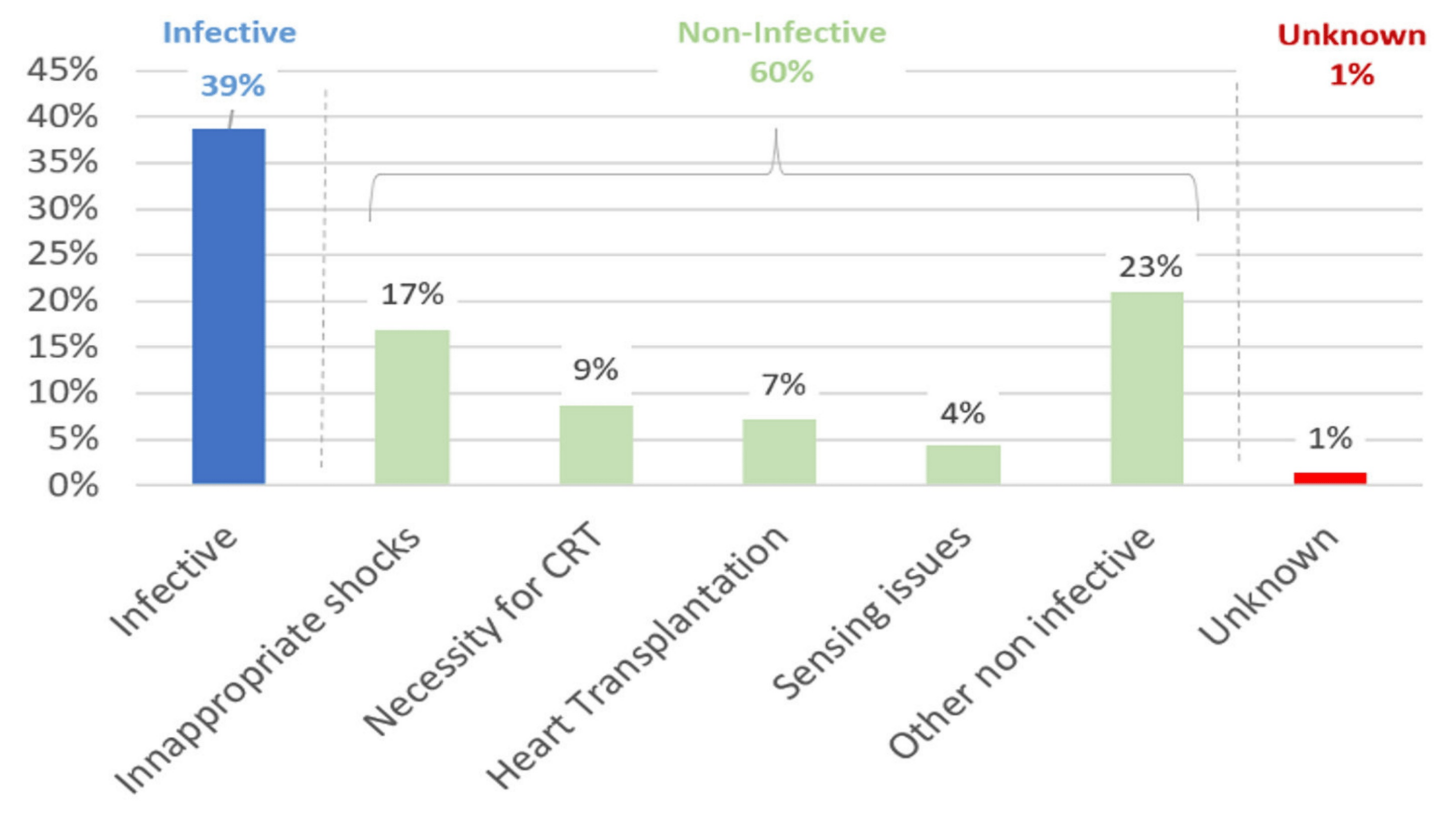
Indications for subcutaneous lead extraction. From ref [8].

Indications for lead extraction include body fractures, infections, or the need for pacing or managing inappropriate shocks through reprogramming (Figure 5).

Behar et al. [11] note that simple traction suffices for 59.4% of cases, with 28.1% requiring specific sheaths to prevent fibrosis, and 9.4% necessitating additional incisions. Infection risks, as reported in various studies, remain notably lower compared to transvenous devices. These outcomes align with the Italian national registry S-ICD Rhythm Detect, involving 2,718 patients, with 71 requiring system extraction. Variations in single traction success rates are observed among studies. De Filippo et al. reported successful extraction in all 71 patients, with 84% managed through manual traction and the remainder needing non-powered mechanical sheaths. Their data highlight a correlation between the timing of explantation and sheath necessity, with median times from implantation of 20 months in successful traction cases and 30 months in unsuccessful cases (p = 0.032) [12].

**Figure 6:**
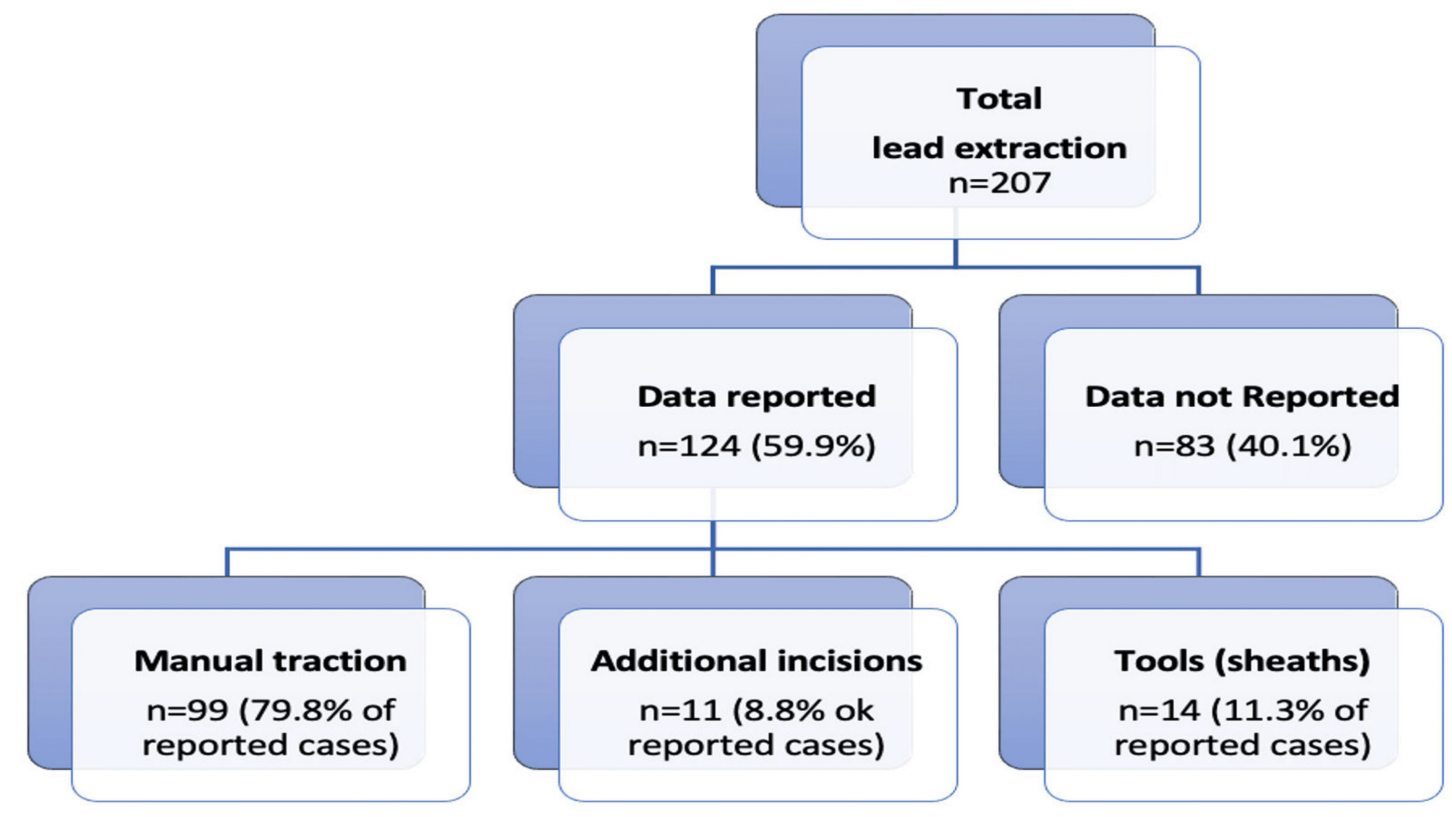
Methods of subcutaneous lead extraction. From ref [8].

Additionally, Riccardio Vio et al. recently conducted a systematic review of S-ICD lead extraction, analyzing 30 studies and 207 patients. Figures 5, 6 illustrate the indications and traction methods from this retrospective review. Their findings show that 60% of extractions were non-infective, predominantly managed with manual traction (similar to De Filippo’s 84% and Behar’s 59.4%). Differences in extraction techniques likely stem from varying explantation timings and indications [8]. Our first case underscores the importance of evaluating all three detection vectors of the S-ICD. An alternate flat vector, elevated impedance, and congruent primary and secondary vectors suggest a subcutaneous lead fracture, as noted in recent recalls. Further studies are warranted to refine treatment strategies based on lead status, whether partial or total fracture. However, consensus is currently lacking, necessitating the ongoing collection of clinical cases to optimize S-ICD lead extraction management.

## Conclusions

S-ICD lead extraction remains infrequent, typically arising from malfunction or infection. Extraction methods vary based on implantation duration and fracture severity. Single traction remains the standard, while preserving coil integrity may necessitate dilator sheaths. Mechanical solutions like the Spectranetics TightRail prove beneficial for complex cases.
